# Effects of Difenoconazole and Imidacloprid Seed Coatings on Soil Microbial Community Diversity and Ecological Function

**DOI:** 10.3390/microorganisms13040806

**Published:** 2025-04-01

**Authors:** Dunfeng Feng, Jiabin Chen, Guo Li, Xiaoying Yang, Yujie Xiong, An Lao, Suzhen Huang, Zheng Zheng

**Affiliations:** 1 Department of Environmental Science and Engineering, Fudan University, Shanghai 200433, China; dffeng22@m.fudan.edu.cn (D.F.); 22210740028@m.fudan.edu.cn (J.C.); guoli21@m.fudan.edu.cn (G.L.); xiaoying@fudan.edu.cn (X.Y.); 22210740080@m.fudan.edu.cn (Y.X.); alao22@m.fudan.edu.cn (A.L.); 2 College of Biological and Environmental Engineering, Zhejiang Shuren University, Hangzhou 310015, China

**Keywords:** pesticide, soil bacteria and fungi, enzyme activity, soil pollution

## Abstract

Difenoconazole and imidacloprid are key components of seed-coating agents, which alter soil microbial community structure and function after application. Existing studies mainly focus on the environmental effects of their spraying application, while research on their impacts on the soil ecosystem when used as seed-coating agents is relatively limited. Through field experiments, this study systematically evaluated and compared the effects of difenoconazole and imidacloprid seed coatings on wheat rhizosphere soil microbial communities and ecological functions by measuring soil enzyme activities, employing 16S rRNA and ITS high-throughput sequencing technologies and predicting KEGG functional pathways. The results showed that imidacloprid and difenoconazole significantly reduced bacterial community diversity, particularly under the high-dosage difenoconazole treatment (0.18 g a.i./kg seed), with a 5.80% decrease in diversity by day 30. This treatment most strongly inhibited the phyla Bacteroidota and Myxococcota, with maximum reductions of 23.87% and 63.57%, respectively. However, the abundance of Actinobacteriota significantly increased, with a maximum increase of 38.53%. Additionally, fungal community diversity significantly increased under both difenoconazole and imidacloprid treatments. Both seed coatings significantly altered the microbial community structure from days 20 to 60, with recovery occurring by day 120. Furthermore, KEGG pathway analysis revealed that the high-dosage difenoconazole treatment (0.18 g a.i./kg seed) significantly activated functional pathways such as cell motility, signal transduction, and membrane transport, whereas the standard dosage (0.12 g a.i./kg seed) exhibited metabolic suppression. This study elucidates the dynamic impacts of seed-coating agent application on soil microbial communities, providing theoretical support for rational pesticide use and the optimization of agricultural strategies.

## 1. Introduction

With the continuous development of global agricultural production, the widespread use of seed-coating pesticides has played a crucial role in increasing crop yields and improving pest and disease control efficiency [1]. However, the application of seed coatings also poses potential threats to non-target soil microbial communities, including alterations in microbial diversity, changes in community structure and function, and potential threats to the long-term health of soil ecosystems [2]. Soil microbial communities are key drivers of material cycling and ecological balance in agricultural ecosystems. Their regulation of biogeochemical cycles, such as those of carbon, nitrogen, and phosphorus, is critical for maintaining soil fertility and plant productivity [3,4]. Although previous studies have explored the effects of pesticides on soil microbes [5,6], they generally focus on short-term effects, such as Sannino et al., who only incubated the pesticide for 1 h after application, observing broad inhibitory effects on glyphosate on phosphatase activities (ranging from 5% to 98%) [7]. Ma et al. investigated the effects of fludioxonil (FL) and a mixture of metalaxyl, fludioxonil, and azoxystrobin (MFA) seed coatings on rhizosphere soil microbes. Their findings showed that treatment with 2.5% FL and 11% MFA significantly increased bacterial Shannon diversity indices at 7 and 21 days, while fungal α-diversity exhibited long-term suppression [2]. A study on the TFC seed coating (10% thifluzamide–fludioxonil–clothianidin) evaluated its impact on the rhizosphere microbiome of wheat seedlings, revealing significant increases in the abundances of Proteobacteria, Actinobacteria, and Patescibacteria among bacteria, and Ascomycota, Mortierellomycota, and Basidiomycota among fungi. These microbes are believed to contribute to plant growth and disease resistance. Conversely, the abundance of Mucoromycota, a fungal group with predominantly pathogenic genera, was reduced [8]. While these studies provide valuable insights, they primarily focus on short-term effects and have not fully explored the long-term, time-dependent dynamics of microbial community changes following the application of seed-coating pesticides. Therefore, this study aims to fill this gap by conducting a long-term (120-day) field experiment to systematically examine the dynamic response characteristics of rhizosphere microbial communities to seed-coating pesticides. This approach will provide crucial insights for optimizing pesticide application strategies and improving the sustainability of agricultural ecosystems. Difenoconazole and imidacloprid are not only efficiently applied as spray pesticides for pest and disease control, but are also widely used as active ingredients in seed coatings for crops such as wheat, peanuts, and corn. Existing research mainly focuses on the environmental effects of difenoconazole and imidacloprid when applied as spray pesticides, while studies on their effects on the soil ecosystem when used as seed coatings are relatively limited. Difenoconazole, a sterol demethylation inhibitor, is extensively used to control various fungal diseases by inhibiting the synthesis of fungal cell membranes. However, in soil environments, it has been confirmed to exert non-target effects on sensitive bacterial and fungal communities [9,10]. A large portion of research on triazole and neonicotinoid insecticides focuses on their toxic effects on insects such as bees. For example, Rei et al. reported that difenoconazole significantly altered fungal composition on the surface of bees and increased the abundance of certain beneficial bacteria [11]. A study on soil microorganisms, conducted by Muñoz-Leoz et al., studied the non-target effects of difenoconazole, deltamethrin, and ethofumesate on soil microbes, reporting significant impacts on multiple soil microbial parameters following the application of 500 mg/kg dry soil pesticides. Among them, difenoconazole had the most pronounced effect on soil microbial parameters. However, these studies focused on respiratory quotients and denitrification potential, rather than changes in microbial community structure [12]. Imidacloprid, a neonicotinoid insecticide, has been shown to exert selective inhibitory effects on certain microbial populations. For instance, its application reduced the total bacterial count in fertile sandy soils [13]. In a 56-day experiment, Cycoń et al. found that imidacloprid negatively affected substrate-induced respiration (SIR), enzyme activity, nitrogen transformation, and bacterial abundance [14]. However, its impact on soil microbial community structure and function remains unclear [15]. We chose difenoconazole and imidacloprid as single-active-ingredient seed coatings because commercially available seed coatings generally use compound formulations to enhance pest and disease control effectiveness, making it difficult to obtain seed coatings with a single active ingredient. Most studies on seed coatings mainly assess the overall ecological effects of compound seed coatings, with limited focus on the specific mechanisms of individual active ingredients. Compound seed coatings typically contain multiple active ingredients, and their complex synergistic or antagonistic effects may obscure the actual impacts of individual components, making it challenging to clearly determine the independent effects of seed coatings on soil microbial communities and ecological functions.

Therefore, this study conducted a long-term (120-day) field experiment in wheat fields by applying different concentrations of difenoconazole and imidacloprid single-active-ingredient seed coatings (standard dosages and increased dosages). Using soil enzyme activity assays, high-throughput sequencing of 16S rRNA and ITS genes, and KEGG functional pathway analysis, we systematically compared and evaluated the effects of difenoconazole and imidacloprid on the rhizosphere soil microbial community and its metabolic functions. The objective was to uncover the potential ecological risks of seed coatings on soil microbes and their possible regulatory mechanisms. The findings provide theoretical support for the rational application of seed coatings and offer important insights for the sustainable management of agricultural ecosystems.

## 2. Materials and Methods

### 2.1. Experimental Site and Design

**Figure 1:**
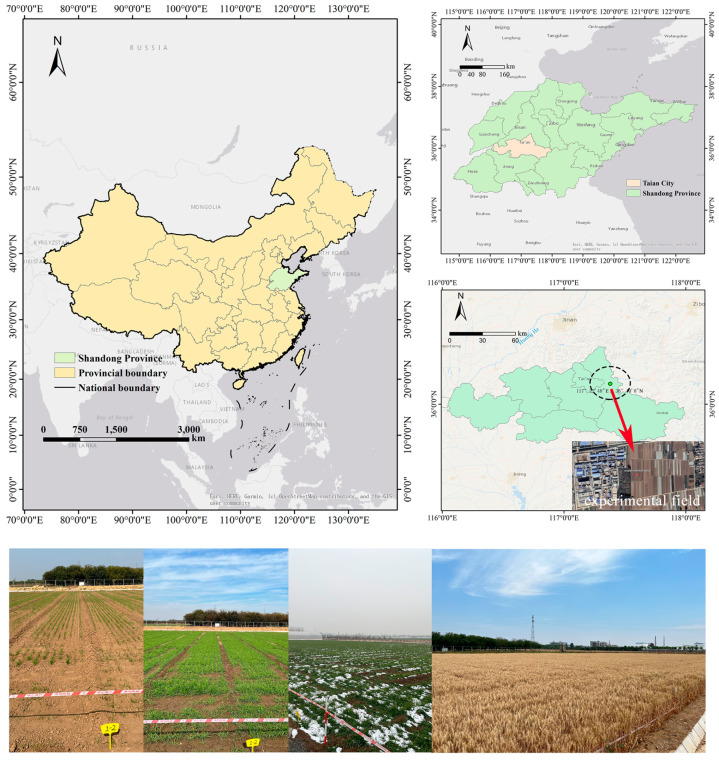
Location of experiment.

The soil type used in the experiment was Haplustalfs, according to the soil taxonomy of the USA [16], and the wheat variety was Jimai 22. The experimental site was located at the Taian Base Experimental Field (Taian, China, 36.10° N, 117.22° E), covering an area of approximately 4000 m^2^ (Figure 1). Based on Köppen climate classification, the region belongs to the Cwa category. The annual average temperature is 14 °C, with the highest temperature in July averaging 26.4 °C and the lowest in January averaging −2.6 °C. The annual average precipitation is 697 millimeters.

Two days before sowing, the experimental field was plowed and leveled. Based on the study by Royer et al. [17], and the recommended application rates of the chemicals, seed-coating agents were applied to the wheat field at two concentrations: the common concentration (D1 and I1) and a concentration 1.5 times higher (D1.5 and I1.5), in addition to a control group (CK). The experiment consisted of 5 groups, with three replicates per group, as shown in Table 1. The seed-coating agents used were 200 g/L difenoconazole (CAS No. 119446-68-3) and 600 g/L imidacloprid (CAS No. 64224-21-1), both purchased from Zhongnong United Biotechnology Co., Ltd., Shandong, China.

Soil samples were collected from each group on days 10, 20, 30, 60, and 120 (from the wheat germination stage D10 to the greening stage D120). The sampling method was conducted as follows: the soil was sampled from five points within a designated area, with samples taken from the surface layer of soil near the wheat roots (depth 0–15 cm). Soil samples were placed in self-sealing bags and labeled. After mixing the soil samples thoroughly, they were pretreated, and all impurities were removed in the laboratory using a 10-mesh screen. A 50 g portion was weighed and filtered through a 40-mesh sieve for enzyme activity measurements. A 10 g portion was added to a sterile centrifuge tube and stored in a super-low-temperature freezer (−80 °C, Thermo Fisher Scientific, Waltham, MA, USA) for subsequent microbiological experiments. The remaining soil was stored in a −20 °C freezer for future use.

### 2.2. Soil Enzyme Activity Assay Methods

The activities of soil catalase, neutral phosphatase, sucrose enzyme, and urease were measured using the corresponding reagent kits (Suzhou Comin Biotechnology Co., Ltd., Suzhou, China). The substrates for measuring catalase, urease, sucrose enzyme, and neutral phosphatase activities were hydrogen peroxide, urea, sucrose and 3,5-dinitrosalicylic acid, and disodium phenyl phosphate, respectively. The absorbance was measured at 240 nm, 578 nm, 510 nm, and 660 nm using a UV-visible spectrophotometer (UV-2600, Shimadzu, Kyoto, Japan), and the enzyme activities of catalase, urease, sucrose enzyme, and neutral phosphatase were calculated based on the formula. Invertase activity was measured according to the methods of Gao et al. [18], while urease, neutral phosphatase, and catalase activities were determined according to the methods of Guan et al. [19] (detailed methods are provided in the Table S1).

### 2.3. Soil Microbial Gene High-Throughput Sequencing and Statistical Analysis Methods

Approximately 0.5 g of soil was weighed, and DNA from different soil samples was extracted using the Mag-Bind Soil DNA Kit (Omega Bio-tek, Norcross, GA, USA). RNA was extracted using the Ribo-Zero rRNA Removal Kit (Illumina, San Diego, CA, USA). The concentrations and purity of the extracted DNA and RNA were measured using a NanoDrop-2000 UV-Vis spectrophotometer (Thermo Fisher Scientific, Waltham, MA, USA). After dilution and aliquoting, the samples were stored at −80 °C in the freezer, awaiting amplicon sequencing. The 16S rRNA gene was amplified using primers 338F (5′-ACTCCTACGGGAGGCAGCAG-3′) and 806R (5′-GGACTACHVGGGTWTCTAAT-3′). To identify fungi, primers ITS1F (5′-CTTGGTCATTTAGAGGAAGTAA-3′) and ITS2R (5′-GCTGCGTTCTTCATCGATGC-3′) were used to amplify the internal transcribed spacer (ITS) region of ribosomal DNA. Further details about the PCR reaction and Illumina sequencing can be found in the article by Daniel Aird et al. [20,21,22]. PCR amplification was performed using primers with barcodes (ABI GeneAmp^®^ 9700 model, Shanghai, China), and sequencing was completed on the Illumina MiSeq platform (Shanghai Meiji Biotechnology Co., Ltd., Shanghai, China). The raw data obtained from amplicon sequencing were first split into individual sample names based on their barcodes, and then sequence clustering was performed using the DADA2 plugin in QIIME (2.0) to generate Amplicon Sequence Variants (ASVs). Sequence correction was applied to obtain operational taxonomic units with single-base precision. The Shannon index was used to describe the alpha diversity of bacterial communities. The Bray–Curtis distance was calculated from the OTU abundance table to measure beta diversity between communities, and non-metric multidimensional scaling (NMDS) was used to visualize differences and potential clustering among samples. Statistical analysis was performed using IBM SPSS 22.0 software and R software (3.5.2) [23,24]. Multi-group comparisons were conducted using Pearson correlation analysis, one-way ANOVA, and Tukey’s post hoc test. The non-parametric Kruskal–Wallis rank sum test was used to identify significant differences (*p* < 0.05). Linear Discriminant Analysis (LDA) effect size measurement was performed to identify significant taxonomic differences between treatments, with an LDA score threshold of 3.5. Taxonomic plots were constructed to describe the different taxa responsible for the differences observed among treatments. To predict microbial community functions, PICRUSt (2.5.2) was used to predict microbial functional profiles. The Kyoto Encyclopedia of Genes and Genomes (KEGG) database was used to normalize, predict, and categorize the generated OTUs according to the online PICRUSt guidelines and KEGG database.

## 3. Results

### 3.1. Soil Enzyme Activity Analysis

**Figure 2:**
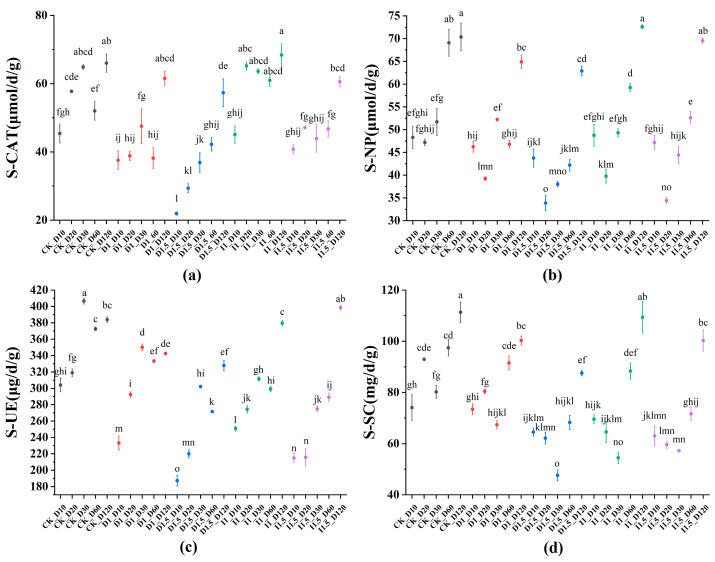
Contents of catalase (**a**), neutral phosphatase (**b**), urease (**c**), and sucrose enzyme (**d**) in the soil of each group. S-CAT refers to soil catalase activity, S-NP refers to soil neutral phosphatase activity, S-UE refers to soil urease activity, and S-SC refers to soil sucrose activity. Different lowercase letters represent a significant difference at *p* < 0.05.

In this study, from wheat seedling emergence (D10) to overwintering (D60), the average catalase activity in the D1, D1.5, and I1.5 groups decreased by 25.83%, 38.67%, and 17.76%, respectively, compared to the CK group, indicating that both seed-coating agents had certain inhibitory effects on the catalase activity in rhizosphere soil (Figure 2a). On day 10, no significant changes were observed in phosphatase activity among the experimental groups. Around day 20, phosphatase activity decreased to the lowest point. The reduction rates in the D1, D1.5, I1, and I1.5 groups were 16.84%, 28.23%, 15.70%, and 27.13%, respectively. Subsequently, phosphatase activity gradually increased. By day 120, the phosphatase activities in the D1.5 and I1.5 groups were still lower than that in the CK group by 7.81% and 10.60%, respectively (Figure 2b). The treatment groups showed varying degrees of inhibition on urease activity in soil from days 10 to 60 compared to the CK group. The D1 and D1.5 treatment groups showed the greatest inhibition on urease activity on day 10, with activities decreasing by 23.26% and 38.40%, respectively. The I1 and I1.5 treatment groups showed the greatest inhibition on day 30, with urease activities decreasing by 23.37% and 32.39%, respectively (Figure 2c). Both seed-coating agents also inhibited sucrose enzyme activity in the soil. On day 30, the D1, D1.5, and I1 groups showed their maximum inhibition, with sucrose enzyme activities decreasing by 16.01%, 40.63%, and 32.10%, respectively. On day 20, the maximum inhibition for the I1.5 group was observed, with sucrose enzyme activity decreasing by 35.89%. By day 120, sucrose enzyme activities in the D1, D1.5, and I1.5 groups were still lower than that in the CK group by 9.93%, 21.32%, and 9.33%, respectively. In the day 120 samples, the enzyme activities of all four enzymes in the I1 and I1.5 groups had recovered to the levels of the CK group (Figure 2d). However, the activities of neutral phosphatase, urease, and sucrose enzyme in the D1 group remained lower than those in the CK group by 7.81%, 10.77%, and 9.93%, respectively. The activities of catalase, neutral phosphatase, urease, and sucrose enzyme in the D1.5 group remained lower than those in the CK group by 13.10%, 10.60%, 14.59%, and 21.32%, respectively.

### 3.2. Changes in Soil Microbial Community Diversity

**Figure 3:**
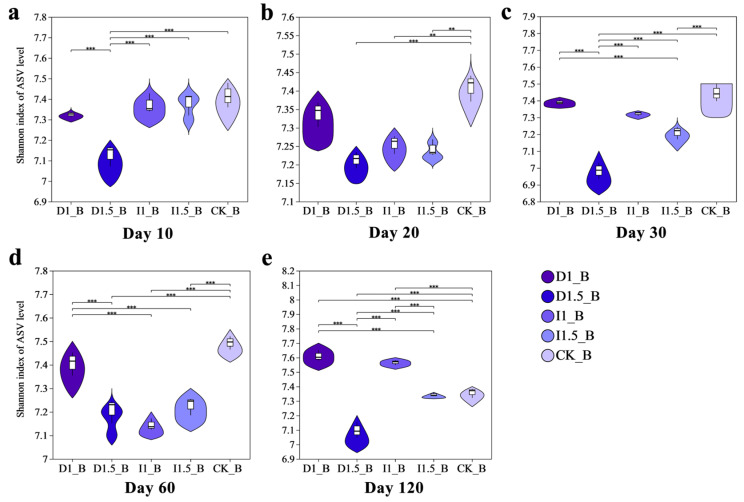
Bacterial Shannon index in wheat rhizosphere soil under treatment with different seed-coating agents at different time points: (**a**) day 10, (**b**) day 20, (**c**) day 30, (**d**) day 60, and (**e**) day 120. ** indicates 0.001 < *p* ≤ 0.01, and *** indicates *p* ≤ 0.001. The uppercase letter ‘B’ represents bacteria throughout the manuscript.

Based on 16S rRNA and ITS sequencing results, the bacterial Shannon diversity index in the D1.5 treatment group decreased by 3.92% compared to that of the control group (CK) at 10 days after sowing in the soil. From days 20 to 60, the bacterial Shannon diversity indices in all treatment groups, except for D1, significantly decreased relative to CK. At 120 days after sowing, the bacterial Shannon diversity index in the D1 group increased by 3.31% compared to that in the CK, while that of the I1.5 group remained 3.50% lower than that of the CK. Changes in imidacloprid dosage had no significant impact on the bacterial Shannon diversity index at 10–20 days. Specifically, the Shannon diversity index in the I1 group decreased by 2.12% at 20 days after sowing, while the I1.5 group showed a 2.21% decrease. However, by 120 days after sowing, the bacterial Shannon diversity index in the I1 group increased by 2.84% compared to that in the CK, and no significant difference was observed between the I1.5 and CK groups (Figure 3).

**Figure 4:**
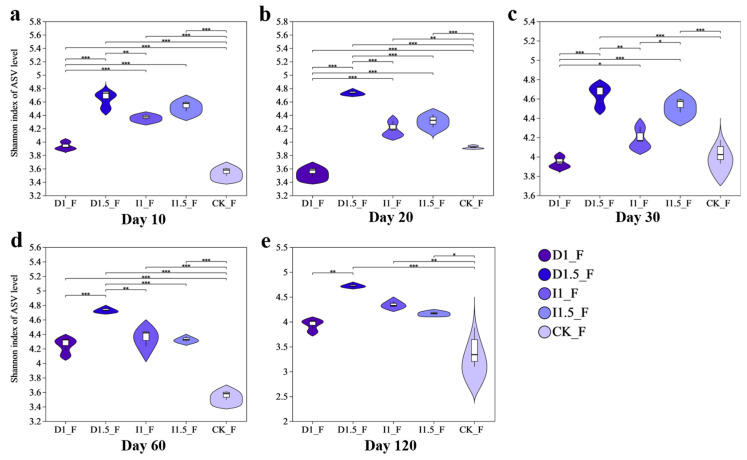
Fungal Shannon index in wheat rhizosphere soil under treatments with different seed-coating agents at different time points: (**a**) day 10, (**b**) day 20, (**c**) day 30, (**d**) day 60, and (**e**) day 120. * indicates 0.01 < *p* ≤ 0.05, ** indicates 0.001 < *p* ≤ 0.01, and *** indicates *p* ≤ 0.001. The uppercase letter ‘F’ represents fungi throughout the manuscript.

Compared to the CK, the fungal Shannon diversity indices in the D1, D1.5, I1, and I1.5 treatment groups all increased, with maximum increments of 20.01%, 35.97%, 25.22%, and 27.63%, respectively (Figure 4). At 10 days after sowing, the fungal Shannon indices in the D1, D1.5, I1, and I1.5 treatment groups significantly increased compared to that of the CK control group, with increases of 14.49%, 35.97%, 25.22%, and 27.63%, respectively (Figure 4a). During days 20–120, the fungal Shannon indices in the D1.5, I1, and I1.5 treatment groups remained significantly higher than that in the CK control group. However, the trend in the D1 treatment group differed from those in the other treatment groups. At 20 days, the fungal Shannon index in the D1 treatment group significantly decreased by 7.80% compared to that in the CK control group (Figure 4b). At 30 and 120 days, the fungal Shannon indices in the D1 treatment group showed no significant differences compared to those in the CK control group (Figure 4c,e). At 60 days, similar to the other treatment groups, the fungal Shannon index in the D1 treatment group significantly increased by 19.44% compared to that in the CK control group (Figure 4d).

### 3.3. Differences in Soil Microbial Community Structure and Composition

**Figure 5:**
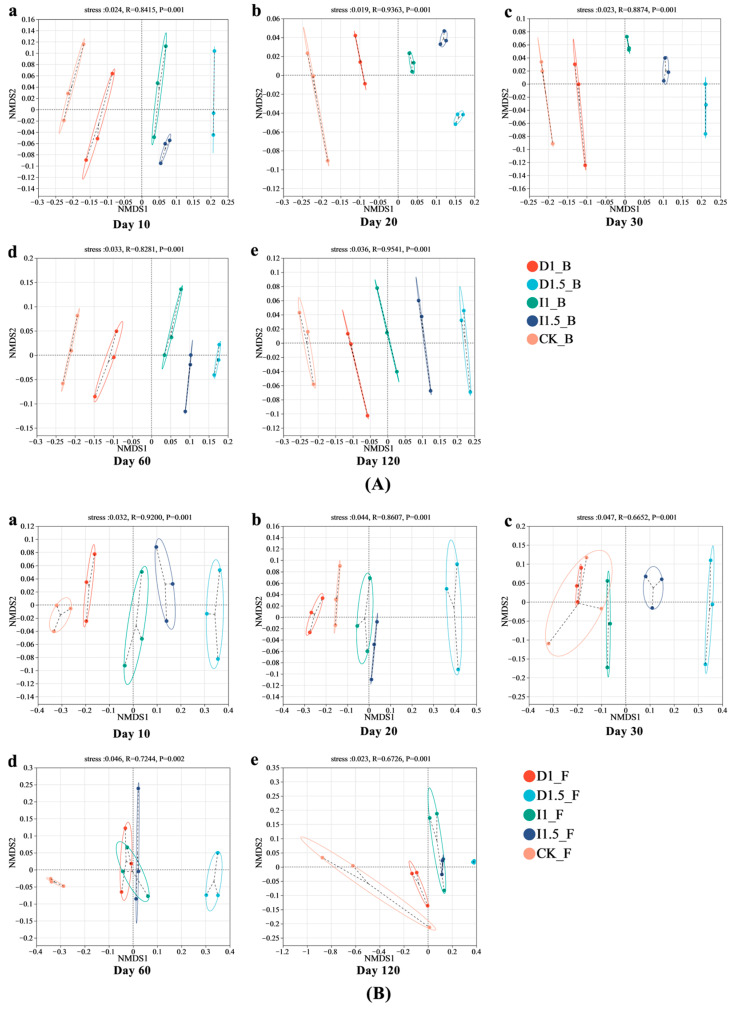
Non-metric multidimensional scaling analysis of bacterial (**A**) and fungal (**B**) communities under treatment with different single seed-coating agents: (**a**) day 10, (**b**) day 20, (**c**) day 30, (**d**) day 60, and (**e**) day 120. The dots in different colors represent samples from different groups, forming grouped ellipses. The closer two sample points are, the more similar the species composition of the two samples. Stress < 0.2 indicates that the plot has explanatory significance.

NMDS analysis based on the Bray–Curtis distance matrix revealed that, at five time points (days 10, 20, 30, 60, and 120), the bacterial community composition in the wheat rhizosphere under different concentrations of difenoconazole and imidacloprid seed-coating treatments significantly differed (*p* < 0.05) from the control group (CK). The magnitude of change in bacterial community composition under the D1 treatment was smaller than that under the I1 treatment, whereas the D1.5 treatment induced significantly greater variation compared to the I1.5 treatment (Figure 5A). The concentrations of seed-coating agents exerted a significant impact on the fungal community. However, after day 60, the differences in fungal community structure between the I1 and I1.5 groups were no longer significant, showing partial overlap in NMDS plots (Figure 5B).

**Figure 6:**
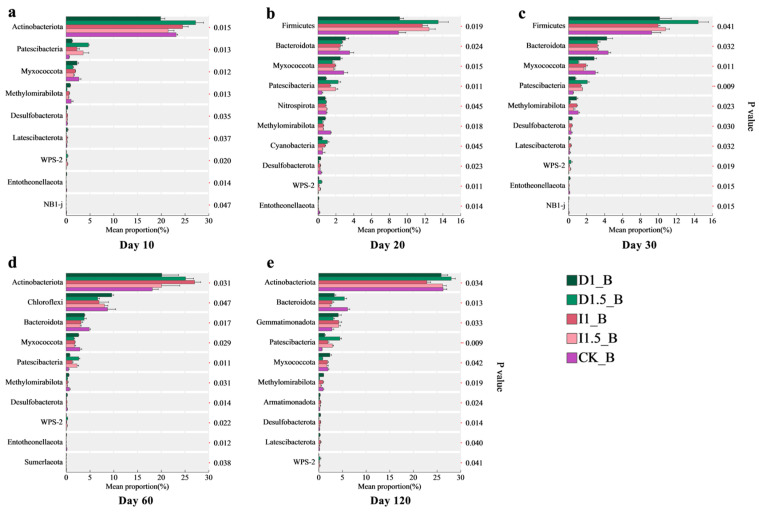
Differential analysis of bacterial composition under different seed-coating treatments at different time points: (**a**) day 10, (**b**) day 20, (**c**) day 30, (**d**) day 60, and (**e**) day 120. The vertical axis represents the names of species at the phylum level, while the horizontal axis indicates the percentage value of species abundance. The rightmost section displays the *p*-values, where * indicates 0.01 < *p* ≤ 0.05, and ** indicates 0.001 < *p* ≤ 0.01.

The compositions of soil rhizosphere bacterial and fungal communities under different treatments were analyzed. The dominant bacterial phyla with significant differences across all treatments included Actinobacteriota, Firmicutes, Bacteroidota, and Chloroflexi (Figure 6). At 10 days after sowing, the relative abundance of Actinobacteriota in the D1 and D1.5 treatment groups decreased by 3.26% and increased by 4.10%, respectively, compared to that in the CK group. In the I1 and I1.5 treatment groups, the relative abundance of Actinobacteriota increased by 1.36% and decreased by 1.69%, respectively, compared to that in the CK. By 60 days, the relative abundance of Actinobacteriota significantly increased in all treatments, with the most pronounced increases observed in the D1.5 and I1 groups (6.99% and 8.89%, respectively). At 120 days, the relative abundance of Actinobacteriota in the D1.5 and I1 groups increased by 1.73% and decreased by 3.44%, respectively, compared to that in the CK.

At 20 and 30 days after sowing, the relative abundance of Firmicutes significantly increased in the D1, D1.5, I1, and I1.5 treatment groups compared to that in the CK. The maximum increase (5.18%) occurred in the D1.5 group at 30 days, followed by the I1.5 group, with a 3.42% increase at 20 days. By 60 days, the relative abundance of Chloroflexi decreased by 2.13%, 1.84%, and 0.71% in the D1.5, I1, and I1.5 groups, respectively, while the D1 group showed a 0.83% increase compared to that in the CK. The abundance of Bacteroidota significantly decreased and remained low until 120 days, with imidacloprid (I-series treatments) exerting a stronger inhibitory effect on Bacteroidota than difenoconazole (D-series treatments). The abundances in the I1 and I1.5 treatment groups decreased by 53.36% and 60.64%, while those in the D1 and D1.5 treatment groups decreased by 45.67% and 11.03%. Although inter-group differences gradually narrowed by 120 days, the relative abundance of Patescibacteria remained significantly higher in all treatment groups than in the CK, with an average increase of 3- to 6-fold.

**Figure 7:**
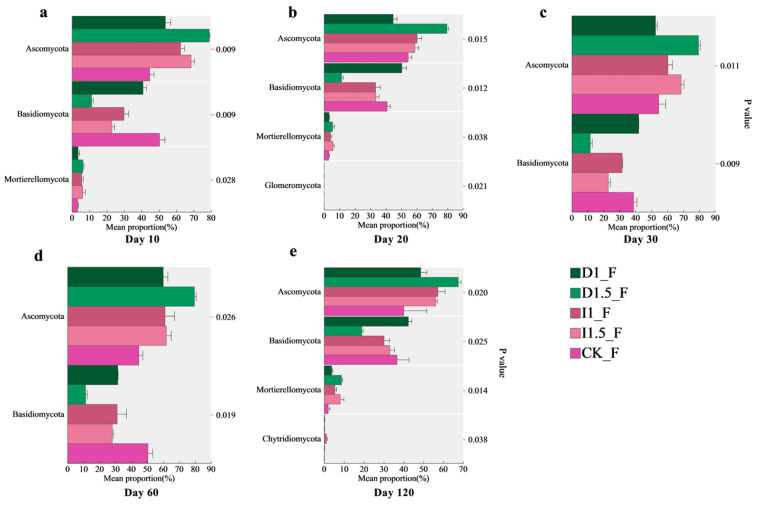
Differential analysis of fungal composition under different seed-coating treatments at different time points: (**a**) day 10, (**b**) day 20, (**c**) day 30, (**d**) day 60, and (**e**) day 120. The vertical axis represents the names of species at the phylum level, while the horizontal axis indicates the percentage value of species abundance. The rightmost section displays the *p*-values, where * indicates 0.01 < *p* ≤ 0.05, and ** indicates 0.001 < *p* ≤ 0.01.

For fungal communities, the dominant phyla with significant differences were Ascomycota, Basidiomycota, and Mortierellomycota (Figure 7). At 10 days after sowing, all treatments increased the relative abundances of Ascomycota and Mortierellomycota, but decreased Basidiomycota compared to those in the CK. These changes were more pronounced in the high-concentration seed-coating treatment groups (D1.5, I1.5) than in the low-concentration groups (D1, I1). In the I1 and I1.5 treatment groups, the relative abundance of Ascomycota increased by 39.65% and 53.24%, while that of Mucoromycota increased by 74.15% and 90.15%. The abundance of Basidiomycota decreased by 40.62% and 54.56%. In the D1 and D1.5 treatment groups, the relative abundance of Ascomycota increased by 20.14% and 76.51%, while that of Mucoromycota increased by 8.36% and 95.73%. The abundance of Basidiomycota decreased by 18.90% and 77.61%. From 20 to 30 days after sowing, the trends for Ascomycota and Basidiomycota persisted in all treatments except D1, with the D1.5 group showing the strongest impact. The relative abundance of Ascomycota increased by 46.20% and 45.78% at days 20 and 30, respectively, while the relative abundance of Basidiomycota decreased by 72.30% and 70.11%, respectively. Imidacloprid treatments (I1, I1.5) significantly reduced the relative abundance of Basidiomycota (7.47% and 7.35% decreases, respectively, at 20 days). Similar patterns were observed at 60 and 120 days, although the differences in effects among different imidacloprid concentration treatments gradually diminished.

### 3.4. Species Enrichment and Microbial Taxa

**Figure 8:**
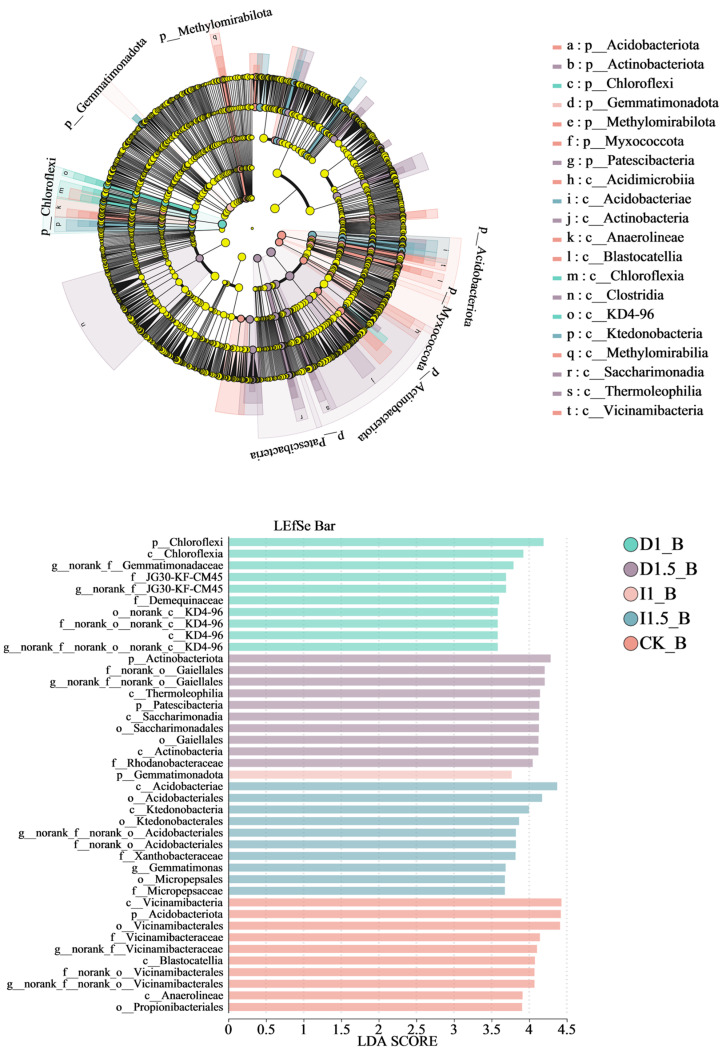
LDA effect size (LEfSe): taxonomic cladogram of bacterial communities under treatment with different seed-coating agents. The nodes of significantly different taxonomic units are colored, while those with no significant differences are shown in light yellow. For each detected taxonomic unit, the corresponding node in the taxonomic tree is colored according to the highest ranked group of the taxon. The bar chart displays bacterial groups with significant differences. The larger the LDA score obtained through linear regression analysis, the greater the impact of species abundance on the differential effect.

In total, 94 distinct bacterial species (Table S2, LDA > 3.5) were identified, with 12 enriched in the D1 treatment, 28 in the D1.5, 1 in the I1, 21 in the I1.5, and 32 in the CK control groups. Figure 8 displays the top 10 species ranked by LDA values for each group. Chloroflexi, Actinobacteriota, Gemmatimonadota, Acidobacteriota, and Vicinamibacteria were the most influential bacterial taxa in the D1, D1.5, I1, I1.5, and CK groups, respectively. Under identical analytical conditions (Figure 8, LDA > 3.5), rhizospheric bacterial species exhibited greater enrichment in the D1.5 treatment and CK control groups, with the enrichment order ranked as CK > D1.5 > I1.5 > D1 > I1.

Linear discriminant analysis effect size (LEfSe, LDA > 3.5) revealed taxonomic composition differences in microbial communities, highlighting shifts in bacterial and fungal communities from the phylum to genus levels across seed-coating treatment groups. In the control group (CK), significant enrichment was observed in Acidobacteriota (phylum to genus), Vicinamibacteria (class to genus), Blastocatellia (class to genus), Methylomirabilota (phylum to genus), Propionibacteriales (order to family), Acidimicrobiia (order to family), and Myxococcota (phylum) taxa, typically associated with soil ecosystem stability. The standard difenoconazole treatment group (D1) showed marked enrichment of Chloroflexi (phylum to genus) and KD4-96 (order to genus), which may correlate with environmental adaptation post-difenoconazole treatment. The elevated difenoconazole group (D1.5) exhibited enrichment of Patescibacteria (phylum to genus), Xanthomonadales (order to genus), Acetobacterales (order to family), and Actinobacteriota (order to genus), suggesting enhanced promotion of specific bacterial taxa under increased treatment. The standard imidacloprid group (I1) demonstrated significant enrichment of Gemmatimonadota (class to family), while the elevated imidacloprid group (I1.5) enriched Acidobacteriae (class to genus) and Ktedonobacteria (class to genus).

**Figure 9:**
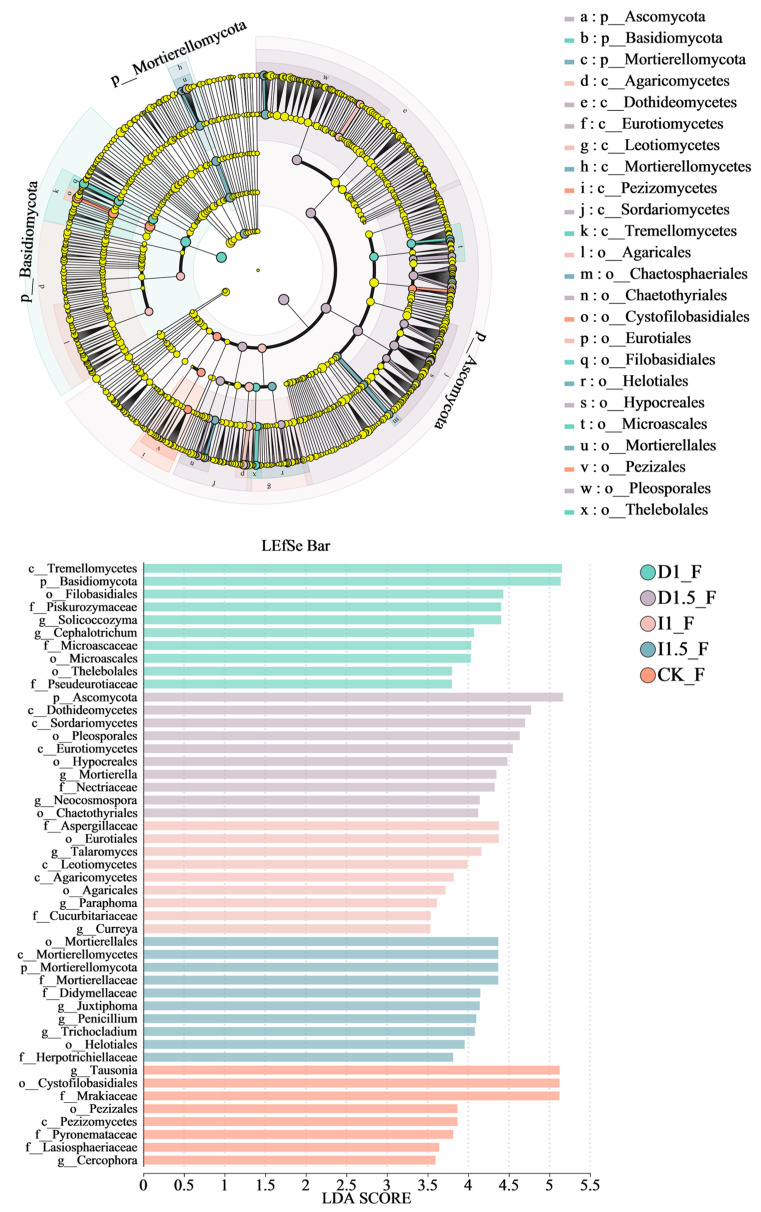
LDA effect size (LEfSe): taxonomic cladogram of fungal communities under treatment with different seed-coating agents. The nodes of significantly different taxonomic units are colored, while those with no significant difference are shown in light yellow. For each detected taxonomic unit, the corresponding node in the taxonomic tree is colored according to the highest ranked group of the taxon. The bar chart displays fungal groups with significant differences. The larger the LDA score obtained through linear regression analysis, the greater the impact of species abundance on the differential effect.

For fungi, 73 distinct species were identified (Table S3, LDA > 3.5), with 11 enriched in the D1, 27 in the D1.5, 11 in the I1, 15 in the I1.5, and 9 in the CK groups. Tremellomycetes, Ascomycota, Aspergillaceae, Mortierellomycetes, and *Tausonia* were the most influential fungal taxa in the D1, D1.5, I1, I1.5, and CK groups, respectively. Under the same analytical conditions (Figure 9, LDA > 3.5), rhizospheric fungal species showed greater enrichment in the D1.5 treatment and I1.5 control groups, ranked as D1.5 > I1.5 > D1 = I1 > CK. The control group (CK) exhibited significant enrichment of Lasiosphaeriaceae (family to genus), Pezizomycetes (class to family), and Cystofilobasidiales (order to genus). The standard difenoconazole group (D1) enriched Thelebolales (order to genus) and *Cephalotrichum* (genus). The elevated difenoconazole group (D1.5) showed notable enrichment of Dothideomycetes (class to order), Chaetomiaceae (family to genus), and Hypocreales (order to genus). The standard imidacloprid group (I1) enriched *Paraphoma* (genus) and Leotiomycetes (class), while the elevated imidacloprid group (I1.5) significantly enriched Juxtiphoma (family to genus), *Trichocladium* (genus), and other fungi.

### 3.5. Soil Microecosystem Stability and Function

According to Table 2, compared to the CK control group, the D1 group showed lower relative abundances in eight pathways: amino acid metabolism (−1.90%); carbohydrate metabolism (−1.52%); energy metabolism (−1.25%); folding, sorting, and degradation (−0.98%); glycan biosynthesis and metabolism (−1.55%); nucleotide metabolism (−1.40%); translation (−0.75%); and transport and catabolism (−3.91%). No significant changes were observed in the relative abundance of the remaining 14 pathways. In contrast, the D1.5 group showed higher abundance in 14 pathways compared to the CK; the most significant increases were observed in cell motility (19.77%), followed by signal transduction (7.93%), membrane transport (5.55%), xenobiotic biodegradation and metabolism (4.84%), and transport and catabolism (4.51%). However, the signaling molecules and interaction pathway and the translation pathway decreased by 23.91% and 0.88%, respectively. The abundance initially decreased and then increased with the increasing concentration of difenoconazole. The I1 group, except for the cell motility pathway, showed an 8.78% increase in relative abundance. The relative abundances of six pathways were lower than those of the CK group, including amino acid metabolism (−1.29%); energy metabolism (−0.79%); folding, sorting, and degradation (−0.97%); glycan biosynthesis and metabolism (−1.78%); signaling molecules and interaction (−18.15%); and translation (−1.17%). The relative abundance of the remaining 15 pathways showed no significant changes. The I1.5 group exhibited an increase in the relative abundance of the cell motility pathway by 12.96% and the signal transduction pathway by 3.48%. The relative abundances of five pathways remained lower than those of the CK group, including amino acid metabolism (−1.34%); energy metabolism (−0.31%); folding, sorting, and degradation (−0.92%); signaling molecules and interaction (−25.27%); and translation (−1.37%). The relative abundance of the remaining 15 pathways showed no significant changes.

## 4. Discussion

### 4.1. Analysis of the Effects of Seed-Coating Agents on Soil Enzyme Activity

Soil enzyme activity affects the cycling of carbon and nitrogen and is used to assess soil pollution levels [25,26]. Many studies have reported the impact of imidacloprid on soil enzyme activity. Singh et al. reported an increase in catalase activity in the soil after planting peanut seeds treated with imidacloprid (2.8 g a.i./kg seed) [27]. Cycoń et al. found that low concentrations of imidacloprid (1 mg/kg soil) inhibited the activity of soil phosphatase and urease, while, at higher concentrations of imidacloprid (10 mg/kg soil), the inhibition of these two enzymes intensified, and catalase activity was also suppressed. They explained that the application of imidacloprid had negative effects on biochemical processes, such as substrate-induced respiration (SIR), and significantly impacted the numbers of nitrifying and nitrogen-fixing bacteria in the soil [14]. In this study, both seed-coating agents reduced the activities of catalase (except for in the I1 group), neutral phosphatase, urease, and sucrase in the soil at the roots of wheat plants at 10–60 days, with higher concentrations of the seed-coating agents showing more significant inhibition. Heterogeneity in soil catalase response to low concentrations of imidacloprid was observed in this study. Catalase activity was not sensitive to the I1 treatment, and the activity in this group at 60 days was higher than that in the CK group. This may be attributed to the “hormesis effect”, in which low-dose pollutants stimulate the microbial stress defense system [28]. When the concentration of imidacloprid is below the threshold, bacterial groups such as Actinobacteria, which produce catalas, may eliminate reactive oxygen species (ROS) through the antioxidant system [29], thereby enhancing enzyme activity. At the same time, soil catalase may undergo oxidation coupling reactions with pesticides [30], leading to the decomposition or inactivation of organic pollutants, thereby reducing the negative impact of pesticides on microbial growth and lowering the extent of pesticide influence on catalase activity. Phosphatase plays an important role in the phosphorus cycle of agricultural ecosystems and is critical for the bioremediation of pesticides [31]. This study found that, at 10 days, there were no significant changes in phosphatase activity in the experimental groups. As an extracellular enzyme, phosphatase is immobilized on colloidal surfaces through adsorption by soil organic matter (e.g., humic acid) and clay minerals, forming enzyme–colloid complexes. These complexes temporarily shield the enzyme from direct contact with pesticide molecules, thereby delaying degradation in the short term [32]. Additionally, the low-concentration difenoconazole group (D1) showed no significant impact on the signaling molecules and interaction pathway during the initial exposure period (10 days), but the signal transduction pathway was already disrupted. This suggests that microorganisms temporarily maintained phosphatase synthesis and secretion functions through functional redundancy. Similarly, although the low-concentration imidacloprid group (I1) was inhibited the signaling molecules and interaction pathway, microorganisms compensated for the communication impairment by enhancing the cell motility pathway, thereby delaying the decline in phosphatase activity. Overall, about 20 days after pesticide application, the activity of phosphatase decreased to a low point. By day 20, difenoconazole had fully exerted its effects, directly interfering with energy metabolism by disrupting microbial membrane structures. The compensatory mechanism (activation of the signal transduction pathway by 7.93% at day 20) proved insufficient to counteract the toxicity, leading to a collapse in phosphatase activity. In the imidacloprid-treated group, dysregulation of the quorum sensing system rendered microorganisms unable to coordinate phosphatase secretion, while increased motility consumed additional energy, indirectly depleting metabolic resources. This period may correspond to the significant release phase of difenoconazole and imidacloprid in the field. Subsequently, the phosphatase activity gradually increased, and the inhibitory effect weakened as the exposure time increased. At 120 days, the phosphatase activity in the D1.5 treatment group was still lower than that in the CK group. Difenoconazole has high persistence and strong chemical stability, which leads to more significant impacts on microbial communities under long-term exposure [33]. A study conducted in the North China Plain reported that the dissipation kinetics equation of difenoconazole in the 20 cm shallow soil layer was *C* = 0.4265 *e*^−0.0135*t*^, with a correlation coefficient of r = 0.8761, and a half-life of 51.3 days [34]. This still indicates the need to focus on the cumulative effect of applying higher concentrations of difenoconazole. Related studies have reported that the application of imidacloprid significantly reduces urease activity in the soil, and this inhibitory effect intensifies with increased application dose and exposure time, peaking at 15 days. At 30 days, the adverse impact of imidacloprid on microbial diversity was still observed [35], though the duration of this effect was not further explored. The results of this study show that, at 120 days, the urease activity in the soil of the I1 and I1.5 groups had recovered to the level of the CK group. Yang et al. reported a study in Shandong Province on the dissipation kinetics of imidacloprid in the shallow soil layer, with the equation *C* = 26.47 *e*^−0.0860*t*^, a correlation coefficient of r = 0.9531, and a half-life of approximately 8.06 days [36]. This supports the finding that the long-term impact of imidacloprid on soil urease activity is lower than that of difenoconazole. Additionally, the degradation products of imidacloprid (such as 6-chloronicotinic acid) may pose persistent secondary toxicity to soil microorganisms, which could be one of the reasons for the delayed peak in inhibitory effects [37]. As urease activity increased, sucrase activity decreased. This relationship may be due to the overlap in metabolic pathways that these two enzymes share when decomposing organic matter in the soil. Urease mainly participates in the breakdown of urea, while sucrase primarily breaks down sucrose. These two enzymes may involve common metabolic pathways during the degradation process, such as glycolysis and the tricarboxylic acid cycle [38,39]. With the degradation of pesticides and their breakdown products serving as nutrients for soil microorganisms, particularly in the imidacloprid treatment group, the impact on soil sucrase activity decreased in the 120-day samples.

### 4.2. Analysis of the Effects of Seed-Coating Agents on the Structure and Diversity of Rhizospheric Soil Microbial Communities

Difenoconazole and imidacloprid caused significant changes in the composition and diversity of microbial populations. Based on 16S rRNA and ITS sequencing results, the change in bacterial community composition and diversity under the D1 treatment was smaller than that under the I1 treatment, while the changes caused by the D1.5 treatment were significantly greater than those caused by the I1.5 treatment. This suggests that different seed-coating agents have significant impacts on bacterial communities, with soil bacterial and fungal communities being more sensitive to the dosage of difenoconazole than to that of imidacloprid. This may be because, under incremental doses of difenoconazole, soil microorganisms were subjected to stronger stress, resulting in significant inhibition or death of sensitive microbial populations, and the recovery ability of the microbial community experienced long-term effects. Difenoconazole, at the D1.5 dosage, significantly reduced the bacterial Shannon index, leading to an irreversible loss of ecological niche. This indicates that, after core microbial populations were suppressed, it was difficult for the community to be re-established or recover through niche filling [40,41]. Imidacloprid, as a new neonicotinoid insecticide, caused non-target inhibition of certain sensitive bacterial species in the soil bacterial community, leading to the reduction or extinction of some populations, thus lowering the overall diversity of the community. The effect of dosage variation on the microbial community was relatively small [42]. Imidacloprid has a significantly lower octanol–water partition coefficient (log Kow = 0.92) [43] compared to difenoconazole (log Kow = 4.36) [44]. The low log Kow of imidacloprid limits its adsorption through hydrophobic interactions, but its electron-deficient pyridine ring can interact with electron-rich aromatic clusters in soil organic matter via electron donor–acceptor (EDA) interactions, leading to local accumulation. However, due to difenoconazole’s strong hydrophobic properties and aromatic π-π interactions, its overall adsorption strength is much higher than that of imidacloprid [45,46]. Therefore, imidacloprid exists in a freer form in the soil, making it more bioavailable, but, due to its strong dilution effect in the aqueous phase, the changes in local concentration are small, resulting in a relatively weaker impact of dose variation on the microbial community. Varying the dosage of imidacloprid had no significant impact on the Shannon index of bacteria at 10–20 days, with the I1 treatment group decreasing by 2.12% on day 20 after sowing, and the I1.5 treatment group decreasing by 2.21%. Li et al. reported that spraying 2 mL of a 100 mg/L high-concentration imidacloprid solution led to a 40% increase in ATP consumption in the rhizosphere soil microorganisms of cabbage seedlings, the accumulation of detoxification byproducts, and the inhibition of oxidative phosphorylation and coenzyme metabolism [47]. In this study, we found that the abundances of the amino acid metabolism, energy metabolism, and glycan biosynthesis and metabolism pathways were downregulated in the I1 treatment group. The inhibition of amino acid metabolism and energy metabolism reduced soil microbial activity and growth capacity, while the suppression of glycan biosynthesis and metabolism affected cell wall synthesis and microbial interactions, thereby impairing microbial survival. This toxicological effect explains the decrease in the bacterial Shannon index. However, on day 120 after sowing, the bacterial Shannon index in the I1 treatment group increased by 2.84% compared to that in the CK group, and the Shannon index of the I1.5 treatment group showed no significant difference from the CK control group (Figure 3). This suggests that the constant imidacloprid seed-coating treatment group might have gradually recovered and enhanced the functional core microbial populations in the later stage. Li et al. also reported the metabolic effects of spraying a low-concentration imidacloprid solution (2 mL, 10 mg/L) on the rhizosphere microorganisms of Chinese cabbage seedlings. They found that the genes for cytochrome P450 (CYP450) and glutathione S-transferase (GST) in microorganisms were upregulated 2–3 times, accelerating imidacloprid degradation. Similar to this study, Actinobacteria and other functional bacteria were enriched in the I1 group, collaborating to counteract the pesticide toxicity [47]. NMDS analysis based on the Bray–Curtis matrix (Figure 5) showed that the impacts of the treatments on bacterial community structure were more persistent than on fungi, which may be due to the selective stress effect of the two chemicals or their degradation products on bacteria, or the alteration of the relative abundances of certain key functional bacteria, thereby delaying the recovery process of the entire bacterial community. Fungi, on the other hand, generally exhibit higher tolerance, especially certain pesticide-resistant fungi that can rapidly occupy ecological niches, leading to a quicker recovery of the fungal community after pesticide degradation [48,49]. Bacterial community structure, due to its higher diversity and more complex ecological networks, is more sensitive to the non-target effects of imidacloprid, and its recovery process is slower. Difenoconazole also exerts non-target effects on the bacterial community, and its dose variations significantly affect both bacterial and fungal communities [5,50]. The D1 treatment had the smallest impact on the rhizospheric soil microbial community structure, suggesting that it may not have reached the threshold concentration necessary to induce more significant structural changes in the microbial population. The D1.5 treatment, however, significantly impacted the rhizospheric fungal community, which was consistent with the trend observed in the bacterial community changes. As a sterol demethylation inhibitor, difenoconazole directly affects the synthesis of fungal cell membranes and should theoretically significantly inhibit soil fungal community diversity, while its effect on bacteria may be smaller. However, experimental results differed from the hypothesis, as the diversity of wheat rhizosphere soil fungal communities increased. This could be due to difenoconazole directly inhibiting certain sensitive bacteria such as Actinobacteria, Basidiomycota, and Methylomirabilota, leading to reduced bacterial diversity and lessening the competitive pressure on some fungi, such as Mortierellomycota, thereby promoting fungal diversity [51]. At the same time, difenoconazole may exhibit non-lethal effects on certain fungi, promoting the relative reproductive advantages of resistant fungi, such as Ascomycota and Mortierellomycota, thus increasing fungal diversity [10]. On day 60 after sowing, the bacterial and fungal community structures still showed significant differences from the control group, indicating that difenoconazole and imidacloprid seed-coating agents, or their degradation products, exert long-term stress on microbial communities. Furthermore, pesticide application may enhance the competitive advantage of certain, more adaptive, fungal populations, making it difficult for the community structure to recover to the control group state.

### 4.3. Analysis of the Effects of Seed-Coating Agents on the Composition of Rhizospheric Soil Microbial Communities

We found significant differences in the dominant bacterial phyla, including Actinobacteriota, Firmicutes, Bacteroidota, and Chloroflexi (Figure 6), among all treatment groups. Actinobacteriota and Bacteroidota contain many key functional microorganisms related to carbon, nitrogen, and phosphorus cycles, while Firmicutes and Chloroflexi participate in the carbon cycle through organic matter decomposition [4,52]. On day 10, the relative abundance of Actinobacteriota was the highest in the D1.5 treatment group, while the D1 treatment group showed the opposite trend. Actinobacteriota might be more sensitive to the constant dose of difenoconazole, leading to mild inhibition. On day 60, although difenoconazole remained an exogenous stress source under the D1.5 treatment, it might have better triggered the tolerance mechanisms or adaptive responses of Actinobacteriota compared to the D1 treatment [53]. Certain species of Actinobacteriota (e.g., *Arthrobacter* sp. and *Microbacterium* sp.) may exhibit stronger resistance or adaptability at this dose [54], leading to an increase in their abundance relative to the control group. The increase in Actinobacteriota abundance might have promoted the active cycling of carbon and nitrogen in the soil, particularly processes related to organic matter decomposition and nutrient transformation [55]. Furthermore, the increase in Firmicutes abundance in the D1, D1.5, I1, and I1.5 treatment groups after 20 and 30 days may be related to their stronger survival abilities under adverse conditions [54], which helps enhance the ecological stability of the soil under stress. However, the decrease in Bacteroidota abundance might reduce soil microbial community diversity and impair organic matter degradation and disease control functions, thus affecting soil fertility and crop growth. The D1 group showed less significant changes in microbial abundance at days 20 and 30, possibly because the constant dose of difenoconazole did not exert strong enough pressure on the soil microbial community, resulting in a mild microbial response. The significance of differences in Firmicutes among different treatment groups decreased before day 60, but the effects of treatments on Actinobacteriota, Bacteroidota, and Patescibacteria continued until day 120. Notably, Patescibacteria may have gained a competitive advantage in the ecological niche in response to external disturbances. Due to their typically minimal genomes and metabolic functions [56], the increase in their abundance may indicate a reduction in the overall metabolic complexity of the soil microbial community. Particularly, the use of incremental doses of difenoconazole reshaped the soil microbial community structure by altering survival competition among sensitive microbial populations, promoting the expansion of more resistant groups. We observed significant differences in the dominant fungal phyla, including Ascomycota, Basidiomycota, and Mortierellomycota (Figure 7), among all treatment groups. The relative abundance of Basidiomycota showed a decreasing trend in all treatment groups except the D1 group, continuing until day 120. The incremental difenoconazole treatment group had the most significant inhibitory effect on Basidiomycota, and the incremental imidacloprid treatment also had a noticeable negative impact on Basidiomycota. The significant decline in Basidiomycota abundance may be related to the selective pressure exerted by these pesticides and the proliferation of resistant populations [57]. Under the influences of imidacloprid and incremental difenoconazole, microbial populations with stronger adaptive abilities (e.g., Ascomycota) occupied more resources or ecological niches, inhibiting the more sensitive groups, such as Basidiomycota. Ascomycota has a broad enzyme spectrum and higher enzyme activity variability, with strong functions in phosphorus and sulfur metabolism. Basidiomycota, which is the only phylum capable of fully degrading lignin, relies on mechanisms such as peroxidases and laccases for this process [58,59]. On day 120, the relative abundance of Olpidiomycota in the treatment groups decreased significantly, showing sensitivity to both seed-coating agents. The relative abundance of Olpidiomycota in the D1, D1.5, I1, and I1.5 treatment groups decreased, from 18.86% in the control group to 2.60%, 0.03%, 3.58%, and 0.54%, respectively (Figure S1). Microbial communities that undergo significant changes due to external environmental disturbances can serve as biological assessment indicators [60,61]. This result suggests that Olpidiomycota can be used as a sensitive indicator phylum, and its abundance change could be used to assess the ecological risks of seed-coating agents.

### 4.4. Effects of Seed-Coating Agents on Species Enrichment and Functional Analysis of the Rhizosphere Soil Microbial Ecosystem

The CK group was able to recruit more bacterial species in the natural environment, while the incremental difenoconazole treatment group maintained a higher bacterial species enrichment ability even after intervention. Specific beneficial bacteria, such as Actinobacteriota, were more strongly enriched under the incremental difenoconazole treatment. Difenoconazole may provide additional functional support to the soil ecosystem by inhibiting the growth of plant pathogens in the rhizosphere, participating in nutrient cycling, and producing extracellular enzymes beneficial for crop production [62,63]. The intervention of seed-coating agents on soil bacterial communities showed significant dose dependency, especially in the incremental treatment groups, where the selective enrichment of specific bacterial populations was more pronounced. Significant changes were also observed in the fungal community across different treatment groups. Tremellomycetes, Ascomycota, Aspergillaceae, Mortierellomycetes, and Tausonia were the most impacted fungal groups in the D1, D1.5, I1, I1.5, and CK groups, respectively. Some Ascomycota (e.g., Aspergillus niger) can improve organic matter decomposition in the soil and help plants absorb important nutrients, such as phosphorus [58,64]. Certain fungi in Mortierellomycetes, such as Mortierella spp., are important beneficial soil fungi that improve soil structure by decomposing organic matter, assist in plant nutrient uptake, and may promote plant growth by symbiosis with plant roots [65,66]. The use of seed-coating agents significantly enriched specific functional microbial groups. The control group could recruit more microbial species in natural conditions, showing higher community diversity, while incremental treatments with seed-coating agents (especially the D1.5 group) significantly suppressed pathogenic microorganisms, while selectively promoting the enrichment of key beneficial microbial groups. These groups include Actinobacteriota and Mortierellomycetes, which provide important functional support to the soil ecosystem through organic matter decomposition, nutrient cycling, pathogen suppression, and symbiosis with plant roots [63,66]. This study revealed the differential regulatory mechanisms of difenoconazole and imidacloprid on soil microbial functions through KEGG metabolic pathway analysis. Notably, both pesticides exhibited inhibitory effects on the signaling molecules and interaction pathways, but the degree of inhibition varied significantly. In the difenoconazole-treated groups, the low-concentration D1 group showed no significant changes in this pathway, but the signal transduction pathway was already affected, suggesting that microbes might temporarily maintain communication functions through functional redundancy. In contrast, the high-concentration D1.5 group displayed a significant inhibition rate of 23.91%, indicating the collapse of compensatory mechanisms [67]. Additionally, difenoconazole exhibited broad-spectrum suppression of fundamental metabolic pathways, such as amino acid metabolism and energy metabolism. In comparison, the imidacloprid-treated groups demonstrated a stronger dose-dependent inhibitory effect, with inhibition rates of 18.15% and 25.27% in the I1 and I1.5 groups, respectively. This suppression pattern suggests that imidacloprid may specifically interfere with microbial interspecies communication by disrupting the quorum sensing system. Notably, the cell motility pathway showed a significant upregulation in the imidacloprid-treated groups, increasing by 8.78% and 12.96% in the I1 and I1.5 groups, respectively. We hypothesize that microbes may compensate for impaired communication by enhancing motility. In contrast, the compensatory mechanism in the difenoconazole-treated groups involved broader activation of metabolic pathways [68,69,70], such as a 7.93% increase in signal transduction and a 5.55% increase in membrane transport in the D1.5 group.

In conclusion, difenoconazole, as a fungicide, directly disrupts microbial membrane structure and energy metabolism, whereas imidacloprid’s neurotoxic mechanism exerts a relatively indirect effect on prokaryotes. Future studies could combine metagenomic analysis to validate the expression changes of quorum sensing-related genes (e.g., the luxR family) [71], thereby clarifying the specific molecular mechanisms underlying these inhibitory effects.

## 5. Conclusions

This study conducted a comparative assessment of changes in soil microbial communities and ecological functions in wheat fields over 120 days, following the applications of different concentrations of difenoconazole and imidacloprid. The findings enhance our understanding of microbial responses to pesticide exposure. Both seed-coating agents significantly altered soil enzyme activity and microbial composition, reducing bacterial diversity while increasing fungal diversity. The stress effect of the difenoconazole seed-coating agent was stronger than that of imidacloprid, and high-concentration treatments led to more pronounced microbial community shifts. Additionally, microbial communities exhibited greater sensitivity to changes in difenoconazole dosage compared to imidacloprid. These effects were also time-dependent, gradually weakening over time. Both pesticides induced persistent changes in bacterial diversity and community structure for up to 120 days, posing a risk to soil ecological integrity. Future research should extend to co-exposure scenarios involving combined pesticide applications, prioritizing the dynamic interactions between pesticide degradation products and microbial communities. Cross-seasonal field trials should also be conducted to determine microbial recovery thresholds. Furthermore, this study lays the foundation for investigating microbial adaptation mechanisms under pesticide stress, encompassing the metabolic plasticity of resistant microbial populations and their pesticide degradation potential. These insights will contribute to optimizing seed-coating agent applications to achieve a balance between crop productivity and soil health preservation.

## Figures and Tables

**Table 1:** Experimental groups and dosage of agents.

Experimental Plot	Seed-Coating Agent	Dosage	Active Ingredient (a.i.) Dosage
		(mL/100 kg Seed)	(g a.i./kg Seed)
D1	Difenoconazole	60	0.18
D1.5	Difenoconazole	90	0.12
I1	Imidacloprid	260	1.56
I1.5	Imidacloprid	390	2.34
CK	/	/	/

“1” represents the conventional application concentration of the seed-coating agent, while “1.5” denotes the increased application concentration.

**Table 2:** Abundance information on wheat rhizosphere bacterial KEGG pathways under treatment with different seed-coating agents.

KEGG Pathway Level 2		Abundance			
	CK	D1	D1.5	I1	I1.5
Amino acid metabolism	7256673 ^a^	7118615 ^b^	7360564 ^a^	7163082 ^b^	7159583 ^b^
Biosynthesis of other secondary metabolites	1375167 ^bc^	1357852 ^c^	1407757 ^a^	1369062 ^bc^	1379274 ^b^
Carbohydrate metabolism	8135689 ^b^	8011875 ^c^	8317365 ^a^	8082431 ^bc^	8101170 ^bc^
Cell growth and death	783259 ^a^	772631 ^a^	785538 ^a^	773148 ^a^	780951 ^a^
Cell motility	805601 ^c^	804390 ^c^	964865 ^a^	876328 ^b^	909981 ^b^
Cellular community—prokaryotes	2049325 ^bc^	2005040 ^c^	2156635 ^a^	2066601 ^b^	2093590 ^b^
Energy metabolism	3943684 ^a^	3894438 ^b^	3982746 ^a^	3912713 ^b^	3931347 ^b^
Folding, sorting, and degradation	1210742 ^a^	1198926 ^b^	1212036 ^a^	1198948 ^b^	1199623 ^b^
Glycan biosynthesis and metabolism	1055889 ^a^	1039489 ^b^	1060784 ^a^	1037137 ^b^	1047892 ^a^
Lipid metabolism	2055865 ^b^	2015625 ^b^	2111053 ^a^	2040242 ^b^	2047598 ^b^
Membrane transport	2519541 ^bc^	2435665 ^c^	2659484 ^a^	2507161 ^bc^	2531247 ^b^
Metabolism of cofactors and vitamins	3734868 ^b^	3687917 ^b^	3823488 ^a^	3724570 ^b^	3742783 ^b^
Metabolism of other amino acids	1422787 ^b^	1401232 ^b^	1458655 ^a^	1408848 ^b^	1419205 ^b^
Metabolism of terpenoids and polyketides	1005282 ^a^	986736 ^a^	1007847 ^a^	986904 ^a^	984575 ^a^
Nucleotide metabolism	2080751 ^b^	2051574 ^c^	2123792 ^a^	2063628 ^bc^	2065391 ^bc^
Replication and repair	2151339 ^b^	2135638 ^b^	2205677 ^a^	2149832 ^b^	2145251 ^b^
Signal transduction	2191250 ^cd^	2158910 ^d^	2364970 ^a^	2233998 ^bc^	2267418 ^b^
Signaling molecules and interaction	415 ^a^	370 ^ab^	316 ^b^	340 ^b^	310 ^b^
Transcription	117598 ^bc^	117113 ^c^	121995 ^a^	118851 ^b^	118796 ^b^
Translation	2472324 ^a^	2453725 ^b^	2450675 ^b^	2443311 ^b^	2438333 ^b^
Transport and catabolism	266919 ^b^	256475 ^c^	278956 ^a^	261494 ^bc^	263378 ^bc^
Xenobiotic biodegradation and metabolism	1750479 ^b^	1716149 ^b^	1835164 ^a^	1746310 ^b^	1770457 ^ab^

The lowercase letters indicate the relationships among the columns. Table S4 in the appendix is the version with standard deviations. The first column represents KEGG (Kyoto Encyclopedia of Genes and Genomes) pathway level 2 functional pathways, which provide an intermediate classification grouping, relating biological functions under broader categories. CK is the blank control group, D represents the difenoconazole seed-coating treatment group, I denotes the imidacloprid seed-coating treatment group, “1” indicates the conventional application concentration of the seed-coating agent, and “1.5” represents the increased application concentration.

## Data Availability

The original contributions presented in this study are included in the article; further inquiries can be directed to the corresponding authors.

## Supplementary Materials

The following supporting information can be downloaded at: https://www.mdpi.com/article/10.3390/microorganisms13040806/s1, Figure S1: The relative abundances, at the phylum level, of fungi on day 120; Table S1: The enzyme activity assay methods involved in this study; Table S2: LDA discrimination results table: Bacteria, LDA > 3.5; Table S3: LDA discrimination results table: Fungi, LDA > 3.5; Table S4: Abundance information of wheat rhizosphere bacterial KEGG pathways under treatment with different seed-coating agents.
