# Supplementary material for: Effects of Difenoconazole and Imidacloprid Seed Coatings on Soil Microbial Community Diversity and Ecological Function
Source: Microorganisms. 2025 Apr 1;13(4):806. doi: 10.3390/microorganisms13040806 (PMC12029232; doi:10.3390/microorganisms13040806)
Supplement: Supplementary file 1 [file microorganisms-13-00806-s001.zip › Table S1.pdf]

Table S1. The enzyme activity assay methods involved in this study

Types of Enzyme Activity	Measurement method
Catalase Activity Assay	Triplicate soil samples (2 g each) are mixed with 40 mL of distilled water, followed by the addition of 5 mL H_2O_2 solution. The mixture is shaken for 20 min. Then, 1 mL of potassium aluminum sulfate is quickly added, and the solution is filtered. Subsequently, 5 mL of 1.5 mol/L sulfuric acid solution is added. The absorbance of the filtrate is measured at 240 nm using a UV microplate. A soil-free control and a substrate-free control are included.
Urease Activity Assay	Triplicate soil samples (5 g each) are placed in a 50 mL conical flask and mixed with 1 mL of toluene. The mixture is left to stand for 15 min. Then, 10 mL of 10% urea solution and 20 mL of citrate buffer (pH 6.7) are added. The sample is incubated at 37°C for 24 h. After incubation, the mixture is centrifuged at 6000 rpm for 10 min. A 1 mL aliquot of the supernatant is transferred to a 50 mL graduated tube, and absorbance is measured at 578 nm. A substrate-free control is included.
Invertase Activity Assay	Triplicate soil samples (5 g each) are placed in a 50 mL conical flask and mixed with 15.0 mL of 8% sucrose solution, 5 mL of pH 5.5 phosphate buffer, and five drops of toluene. The mixture is shaken and incubated at 37°C for 24 h. After incubation, the mixture is centrifuged at 6000 rpm for 10 min. A 1 mL aliquot of the supernatant is transferred to a 50 mL colorimetric tube, and absorbance is measured at 510 nm. Each soil sample includes a substrate-free control and a soil-free control.
Neutral Phosphatase Activity Assay	Triplicate soil samples (5 g each) are placed in a 50 mL conical flask and mixed with 2.5 mL of toluene, followed by gentle shaking for 15 min. Then, 20 mL of 0.5% disodium phenyl phosphate (prepared with citrate buffer) is added. After thorough mixing, the sample is incubated at 37°C for 24 h. After incubation, 100 mL of 0.3% aluminum sulfate solution is added to the culture solution, and the mixture is filtered. The filtrate is centrifuged at 6000 rpm for 5 min. A 3 mL aliquot of the supernatant is transferred to a 50 mL volumetric flask, followed by the addition of 5 mL of buffer and 0.2 mL of chlorinated dibromoquinoneimine reagent for color development. The solution is diluted to volume, and after 30 min, absorbance is measured at 660 nm using a spectrophotometer. A soil-free tube and a standard tube are also included.
