# Supplementary material for: Effects of Difenoconazole and Imidacloprid Seed Coatings on Soil Microbial Community Diversity and Ecological Function
Source: Microorganisms. 2025 Apr 1;13(4):806. doi: 10.3390/microorganisms13040806 (PMC12029232; doi:10.3390/microorganisms13040806)
Supplement: Supplementary file 1 [file microorganisms-13-00806-s001.zip › Table S2.pdf]

Table S2. LDA discrimination results table.

Species name	group	Mean	LDA value	P_value
p__Actinobacteriota.c__Acidimicrobiia		4.30	3.59	0.00
p__Methylomirabilota		4.40	3.61	0.00
p__Actinobacteriota.c__Actinobacteria.o_Propionibacteriales.f_Nocardioidaceae		4.02	3.60	0.00
p__Chloroflexi.c__Anaerolineae		4.49	3.85	0.00
p__Acidobacteriota.c__Vicinamibacteria.o__Vicinamibacterales.f__norank_o__Vicinamibacterales		4.58	3.92	0.00
p__Bacteroidota.c__Bacteroidia.o__Cytophagales		4.30	3.91	0.00
p__Chloroflexi.c__Anaerolineae.o__SBR1031.f__A4b.g__norank_f__A4b		4.39	3.79	0.00
p__Proteobacteria.c__Gammaproteobacteria.o_Steroidobacterales.f_Steroidobacteraceae		4.57	4.07	0.00
p__Acidobacteriota.c__Vicinamibacteria		4.05	3.61	0.00
p__Proteobacteria.c__Alphaproteobacteria.o__Azospirillales		3.92	3.52	0.00
p__Acidobacteriota.c__Vicinamibacteria.o__Vicinamibacterales.f__norank_o__Vicinamibacterales.g__norank_f__norank_o__Vicinamibacterales		3.88	3.52	0.00
p__Acidobacteriota.c__Blastocatellia.o_Pyrinomonadales.f__Pyrinomonadaceae.g _ RB41	CK_B	4.03	3.60	0.00
p__Methylomirabilota.c__Methylomirabilia		4.33	3.59	0.00
p__Chloroflexi.c__Anaerolineae.o__SBR1031		4.86	4.43	0.00
p__Chloroflexi.c__Anaerolineae.o__SBR1031.f__A4b		3.94	3.60	0.00
p__Acidobacteriota.c__Vicinamibacteria.o__Vicinamibacterales		4.13	3.66	0.00
p__Methylomirabilota.c__Methylomirabilia.o_Rokubacteriales.f_norank_o__Rokubacteriales.g__norank_f__norank_o__Rokubacteriales		4.03	3.64	0.00
p__Actinobacteriota.c__Acidimicrobiia.o__Microtrichales		4.18	3.82	0.00
p__Proteobacteria.c__Alphaproteobacteria.o__Rhizobiales.f__Beijerinckiaceae.g__Microvirga		4.57	4.07	0.00
p__Myxococcota		4.27	3.88	0.00
p__Acidobacteriota.c__Blastocatellia		4.01	3.54	0.00
p__Acidobacteriota.c__Blastocatellia.o_Pyrinomonadales.f__Pyrinomonadaceae		4.02	3.60	0.00

p__Actinobacteriota.c__Actinobacteria.o__Propionibacteriales		3.96	3.52	0.00
p__Acidobacteriota.c__Vicinamibacteria.o__Vicinamibacterales.f__Vicinamibacteraceae.g__norank_f__Vicinamibacteraceae		5.41	4.29	0.00
p__Acidobacteriota.c__Vicinamibacteria.o__Vicinamibacterales.f__Vicinamibacteraceae		4.90	4.14	0.00
p__Acidobacteriota.c__Blastocatellia.o__Pyrinomonadales		4.31	3.69	0.00
p__Proteobacteria.c__Gammaproteobacteria.o__Steroidobacterales		4.14	3.74	0.00
p__Methylomirabilota.c__Methylomirabilia.o__Rokubacteriales		3.97	3.58	0.00
p__Proteobacteria.c__Alphaproteobacteria.o__Rhizobiales.f__Beijerinckiaceae		5.03	4.19	0.00
p__Methylomirabilota.c__Methylomirabilia.o_Rokubacteriales.f_norank_o__Rokubacteriales		4.84	4.41	0.00
p__Acidobacteriota		3.99	3.51	0.00
p__Chloroflexi.c__Chloroflexia.o__Chloroflexales		3.99	3.68	0.00
p__Chloroflexi.c__Chloroflexia		3.92	3.52	0.00
p__Gemmatimonadota.c__Gemmatimonadetes.o_Gemmatimonadales.f_Gemmatimonadaceae.g_norank_f_Gemmatimonadaceae		3.93	3.52	0.00
p__Chloroflexi.c__Chloroflexia.o__Chloroflexales.f__Roseiflexaceae		4.74	4.21	0.00
p__Chloroflexi.c__Chloroflexia.o__Thermomicrobiales.f _ JG30-KF-CM45		3.97	3.55	0.00
p__Chloroflexi		3.90	3.59	0.00
p__Chloroflexi.c__KD4-96.o__norank_c _ KD4-96		4.03	3.64	0.00
p__Chloroflexi.c__KD4-96.o__norank_c__KD4-96.f__norank_o__norank_c _ KD4-96	D1_B	3.95	3.54	0.00
p__Actinobacteriota.c__Actinobacteria.o__Micrococcales.f__Demequinaceae		4.74	4.21	0.00
p__Chloroflexi.c _ KD4-96		3.99	3.61	0.00
p__Chloroflexi.c__KD4-96.o__norank_c__KD4-96.f__norank_o__norank_c__KD4-96.g__norank_f__norank_o__norank_c__KD4-96		4.13	3.66	0.00
p__Chloroflexi.c__Chloroflexia.o__Thermomicrobiales.f_JG30-KF-CM45.g_norank_f__ JG30-KF-CM45		4.43	3.83	0.00

p__Chloroflexi.c__Chloroflexia.o__Chloroflexales.f_Roseiflexaceae.g_norank_f_Roseiflexaceae		4.61	3.77	0.00
p__Bacteroidota.c_Bacteroidia.o__Chitinophagales		4.51	4.08	0.00
p__Firmicutes.c__Clostridia		3.99	3.67	0.00
p__Patescibacteria.c__Saccharimonadia.o__Saccharimonadales.f _ LWQ8		4.22	3.58	0.00
p__Proteobacteria.c__Alphaproteobacteria.o_Acetobacterales.f_Acetobacteraceae		4.03	3.64	0.00
p__Actinobacteriota		4.27	3.88	0.00
p__Actinobacteriota.c__Thermoleophilia		4.55	3.91	0.00
p__Bacteroidota.c__Bacteroidia.o__Chitinophagales.f_Chitinophagaceae.g_norank_f_Chitinophagaceae		3.96	3.52	0.00
p__Proteobacteria.c__Gammaproteobacteria.o_Burkholderiales.f_Burkholderiaceae		4.06	3.63	0.00
p__Actinobacteriota.c__Thermoleophilia.o__Gaiellales.f__norank_o__Gaiellales		4.22	3.58	0.00
p__Acidobacteriota.c__Acidobacteriae.o__Acidobacteriales.f__Acidobacteriaceae_Subgroup_1		4.53	4.17	0.00
p__Actinobacteriota.c__Thermoleophilia.o__Gaiellales.f__norank_o__Gaiellales.g__norank_f__norank_o__Gaiellales	D1.5_B	4.48	4.10	0.00
p__Proteobacteria.c__Alphaproteobacteria.o__Acetobacterales		4.51	4.14	0.00
p__Proteobacteria.c__Gammaproteobacteria.o__Xanthomonadales.f__Rhodanobacteraceae		4.27	3.88	0.00
p__Proteobacteria.c__Gammaproteobacteria.o__Burkholderiales.f_Burkholderiaceae.g_Burkholderia-Caballeronia-Paraburkholderia		4.24	3.87	0.00
p__Patescibacteria		3.90	3.54	0.00
p__Actinobacteriota.c__Actinobacteria		4.41	4.05	0.00
p__Actinobacteriota.c__Thermoleophilia.o__Gaiellales		3.90	3.56	0.00
p__Actinobacteriota.c__Actinobacteria.o__Frankiales		4.51	4.14	0.00
p__Patescibacteria.c__Saccharimonadia.o__Saccharimonadales.f__WWH38.g__norank_f _ WWH38		5.21	4.12	0.02
p__Proteobacteria.c__Gammaproteobacteria.o__Xanthomonadales		4.79	4.12	0.00
p__Patescibacteria.c__Saccharimonadia.o__Saccharimonadales.f _ WWH38		1.32	3.60	0.02

p__Patescibacteria.c__Saccharimonadia.o__Saccharimonadales.f__LWQ8.g__norank_f _ LWQ8		4.82	4.37	0.00
p__Patescibacteria.c__Saccharimonadia		4.13	3.66	0.00
p__Patescibacteria.c__Saccharimonadia.o__Saccharimonadales		4.38	3.92	0.00
p__Proteobacteria.c__Gammaproteobacteria.o__Xanthomonadales.f__Rhodanobacteraceae.g__Chujaibacter		4.02	3.60	0.00
p__Proteobacteria.c__Gammaproteobacteria.o__Burkholderiales.f__Oxalobacteraceae.g__Massilia		4.22	3.58	0.00
p__Actinobacteriota.c__Actinobacteria.o__Micrococcales.f__Micrococcaceae.g__Arthrobacter		4.02	3.72	0.00
p__Bacteroidota.c__Bacteroidia.o__Chitinophagales.f__Chitinophagaceae		4.58	3.94	0.00
p__Gemmatimonadota	I1_B	4.02	3.72	0.00
p__Proteobacteria.c__Alphaproteobacteria.o__Elsterales.f__norank_o__Elsterales	I1.5_B	3.94	3.60	0.00
p__Acidobacteriota.c__Acidobacteriae.o__Solibacterales		4.49	4.13	0.00
p__Acidobacteriota.c__Acidobacteriae.o__Acidobacteriales.f__norank_o__Acidobacteriales.g__norank_f__norank_o__Acidobacteriales		4.18	3.82	0.00
p__Proteobacteria.c__Alphaproteobacteria.o__Micropepsales		4.49	4.13	0.00
p__Acidobacteriota.c__Acidobacteriae.o__Acidobacteriales.f__unclassified_o__Acidobacteriales.g__unclassified_o__Acidobacteriales		4.15	3.69	0.00
p__Chloroflexi.c__Ktedonobacteria.o__Ktedonobacterales.f _ JG30-KF-AS9		4.08	3.54	0.00
p__Acidobacteriota.c__Acidobacteriae.o__Solibacterales.f__Solibacteraceae		4.06	3.75	0.00
p__Proteobacteria.c__Alphaproteobacteria.o__Micropepsales.f__Micropepsaceae.g__norank_f__Micropepsaceae		4.33	3.88	0.00
p__Chloroflexi.c__Ktedonobacteria.o__Ktedonobacterales.f__JG30-KF-AS9.g__norank_f _ JG30-KF-AS9		3.93	3.52	0.00
p__Proteobacteria.c__Alphaproteobacteria.o__Elsterales		3.97	3.55	0.00
p__Acidobacteriota.c__Acidobacteriae.o__Acidobacteriales		3.87	3.55	0.00
p__Chloroflexi.c__Ktedonobacteria.o__Ktedonobacterales		5.14	4.42	0.00
p__Acidobacteriota.c__Acidobacteriae		4.03	3.60	0.00

p__Acidobacteriota.c__Acidobacteriae.o_Solibacterales.f_Solibacteraceae.g__Candidatus_Solibacter	4.22	3.58	0.00
p__Acidobacteriota.c__Acidobacteriae.o_Acidobacteriales.f_norank_o_Acidobacteriales	4.05	3.58	0.00
p__Gemmatimonadota.c__Gemmatimonadetes.o_Gemmatimonadales.f_Gemmatimonadaceae.g_Gemmatimonas	4.40	3.60	0.00
p__Acidobacteriota.c__Acidobacteriae.o_Acidobacteriales.f_unclassified_o_Acidobacteriales	4.42	4.00	0.00
p__Proteobacteria.c__Alphaproteobacteria.o__Elsterales.f__norank_o__Elsterales.g__norank_f__norank_o__Elsterales	3.99	3.68	0.00
p__Chloroflexi.c__Ktedonobacteria	4.56	3.82	0.00
p__Proteobacteria.c__Alphaproteobacteria.o__Micropepsales.f__Micropepsaceae	4.31	3.69	0.00
p__Proteobacteria.c__Alphaproteobacteria.o__Rhizobiales.f__Xanthobacteraceae	4.00	3.54	0.00

[1] (Bacteria, LDA > 3.5)
