# Supplementary material for: Effects of Difenoconazole and Imidacloprid Seed Coatings on Soil Microbial Community Diversity and Ecological Function
Source: Microorganisms. 2025 Apr 1;13(4):806. doi: 10.3390/microorganisms13040806 (PMC12029232; doi:10.3390/microorganisms13040806)
Supplement: Supplementary file 1 [file microorganisms-13-00806-s001.zip › Table S3.pdf]

Table S3. LDA discrimination results table.

Species name	group	Mean	LDA value	P_value
p_Ascomycota.c_Pezizomycetes.o_Pezizales	CK_F	4.30	3.87	0.01
p_Ascomycota.c_Pezizomycetes.o__Pezizales.f__Pyronemataceae.g__unclassified_f__Pyronemataceae	CK_F	4.21	3.81	0.05
p__Ascomycota.c__Pezizomycetes	CK_F	4.30	3.87	0.01
p_Basidiomycota.c__Tremellomycetes.o_Cystofilobasidiales	CK_F	5.50	5.12	0.00
p_Ascomycota.c_Sordariomycetes.o_Sordariales.f__Lasiosphaeriaceae.g_Cercophora	CK_F	3.99	3.60	0.00
p_Ascomycota.c_Pezizomycetes.o_Pezizales.f_Pyronemataceae	CK_F	4.25	3.81	0.05
p_Basidiomycota.c__Tremellomycetes.o_Cystofilobasidiales.f_Mrakiaceae	CK_F	5.50	5.12	0.00
p_Basidiomycota.c__Tremellomycetes.o_Cystofilobasidiales.f_Mrakiaceae.g__Tausonia	CK_F	5.50	5.13	0.00
p_Ascomycota.c_Sordariomycetes.o__Sordariales.f__Lasiosphaeriaceae	CK_F	4.11	3.64	0.01
p__Ascomycota.c__Sordariomycetes.o__Microascales	D1_F	4.55	4.03	0.00
p_Ascomycota.c_Sordariomycetes.o__Microascales.f__Microascaceae	D1_F	4.54	4.04	0.00
p__Basidiomycota	D1_F	5.63	5.14	0.00
p_Basidiomycota.c_Tremellomycetes.o_Filobasidiales	D1_F	5.03	4.43	0.00
p_Basidiomycota.c_Tremellomycetes.o_Filobasidiales.f_Piskurozymaceae	D1_F	4.99	4.40	0.00
p_Ascomycota.c_Sordariomycetes.o__Microascales.f__Microascaceae.g__Cephalotrichum	D1_F	4.39	4.07	0.00

p__Basidiomycota.c__Tremellomycetes.o_Filobasidiales.f_Piskurozymaceae.g__Solicoccozyma		4.99	4.40	0.00
p_Basidiomycota.c_Tremellomycetes		5.62	5.15	0.00
p__Ascomycota.c__Leotiomycetes.o__Thelebolales.f__Pseudeurotiaceae.g__Pseudogymnoascus		4.18	3.77	0.02
p__Ascomycota.c__Leotiomycetes.o__Thelebolales.f__Pseudeurotiaceae		4.21	3.80	0.01
p__Ascomycota.c__Leotiomycetes.o Thelebolales		4.22	3.80	0.01
p__Ascomycota.c__Sordariomycetes.o__Hypocreales.f__Stachybotryaceae		3.91	3.54	0.02
p__Ascomycota.c__Dothideomycetes.o__Pleosporales.f__unclassified_o__Pleosporales.g__unclassified_o__Pleosporales		4.71	4.37	0.00
p_Ascomycota.c_Sordariomycetes.o_Sordariales.f_Chaetomiaceae.g_Chaetomium		4.27	3.86	0.00
p_Ascomycota.c_Eurotiomycetes.o_Chaetothyriales.f_Trichomeriaceae		3.99	3.66	0.00
p__Ascomycota.c__Dothideomycetes.o__Pleosporales		5.29	4.63	0.00
p_Ascomycota.c_Sordariomycetes.o__Hypocreales.f__Nectriaceae.g__Neocosmospora	D1.5_F	4.66	4.14	0.00
p_Ascomycota.c_Eurotiomycetes.o_Chaetothyriales.f_Trichomeriaceae.g_Knufia		3.99	3.65	0.00
p__Ascomycota.c__Leotiomycetes.o_Helotiales.f_Helotiales_fam_Incertae_sedis.g__Mycoarthris		4.05	3.69	0.00
p__Ascomycota.c__Leotiomycetes.o_Helotiales.f_Helotiales_fam_Incertae_sedis		4.06	3.70	0.00
p__Ascomycota.c__Eurotiomycetes		5.04	4.55	0.00

p__Ascomycota.c__Sordariomycetes.o__Hypocreales.f__Nectriaceae.g__Fusarium	4.69	4.09	0.00
p_Ascomycota.c_Sordariomycetes.o_Hypocreales.f_Nectriaceae	5.02	4.32	0.00
p_Ascomycota.c_Eurotiomycetes.o_Chaetothyriales	4.57	4.12	0.00
p__Ascomycota	5.88	5.17	0.00
p__Ascomycota.c__Sordariomycetes.o__Hypocreales	5.20	4.48	0.00
p__Ascomycota.c__Sordariomycetes.o__Microascales.f__Graphiaceae.g__Graphium	3.89	3.55	0.00
p_Ascomycota.c_Sordariomycetes.o__Sordariales.f__Chaetomiaceae	4.83	4.02	0.03
p_Ascomycota.c_Sordariomycetes	5.55	4.70	0.01
p_Mortierellomycota.c_Mortierellomycetes.o_Mortierellales.f_Mortierellaceae.g_Mortierella	4.81	4.35	0.00
p_Ascomycota.c_Sordariomycetes.o__Microascales.f__Microascaceae.g__Acaulium	3.94	3.57	0.00
p_Ascomycota.c_Sordariomycetes.o_Sordariales.f_Chaetomiaceae.g_Condenascus	4.49	4.04	0.00
p_Ascomycota.c_Sordariomycetes.o_Microascales.f_Graphiaceae	3.89	3.55	0.00
p_Ascomycota.c_Dothideomycetes	5.40	4.77	0.01
p_Ascomycota.c_Sordariomycetes.o__Hypocreales.f__Bionectriaceae	4.43	3.95	0.01
p_Ascomycota.c_Dothideomycetes.o_Pleosporales.f_unclassified_o_Pleosporales	4.71	4.37	0.00
p_Ascomycota.c_Sordariomycetes.o_Hypocreales.f_Stachybotryaceae.g_Albifimbria	3.91	3.56	0.01
p_Ascomycota.c_Sordariomycetes.o_Hypocreales.f_Bionectriaceae.g_Clonostachys	4.04	3.54	0.01

p__Ascomycota.c__Eurotiomycetes.o__Eurotiales.f__Aspergillaceae		4.85	4.38	0.00
p_Ascomycota.c_Dothideomycetes.o_Pleosporales.f_Phaeosphaeriaceae.g_Paraphoma		3.90	3.61	0.04
p_Ascomycota.c_Eurotiomycetes.o_Eurotiales		4.85	4.38	0.00
p__Basidiomycota.c__Agaricomycetes.o__Agaricales		4.11	3.72	0.01
p__Ascomycota.c__Dothideomycetes.o_Pleosporales.f_Cucurbitariaceae		3.98	3.54	0.03
p__Basidiomycota.c__Agaricomycetes		4.26	3.82	0.00
p__Ascomycota.c__Leotiomycetes.o_Helotiales.f_unclassified_o_Helotiales	I1_F	4.00	3.60	0.00
p__Ascomycota.c__Eurotiomycetes.o__Eurotiales.f__Aspergillaceae.g_Talaromyces		4.53	4.16	0.00
p__Ascomycota.c__Leotiomycetes.o_Helotiales.f_unclassified_o_Helotiales.g_unclassified_o_Helotiales		4.00	3.60	0.00
p__Ascomycota.c__Leotiomycetes		4.49	3.99	0.00
p_Ascomycota.c_Dothideomycetes.o_Pleosporales.f_Cucurbitariaceae.g_Curreya		3.98	3.53	0.03
p_Ascomycota.c_Dothideomycetes.o_Pleosporales.f_Didymellaceae.g_Juxtiphoma		4.81	4.14	0.00
p_Mortierellomycota.c_Mortierellomycetes.o_Mortierellales.f_Mortierellaceae.g_Linnemannia		3.87	3.59	0.00
p_Ascomycota.c_Sordariomycetes.o_Chaetosphaeriales.f_Chaetosphaeriaceae	I1.5_F	4.08	3.72	0.00
p_Mortierellomycota.c_Mortierellomycetes.o_Mortierellales		4.84	4.37	0.00
p_Ascomycota.c_Dothideomycetes.o__Pleosporales.f__Didymellaceae		4.96	4.15	0.03

p__Mortierellomycota.c__Mortierellomycetes	4.84	4.37	0.00
p_Ascomycota.c_Eurotiomycetes.o_Chaetothyriales.f_Herpotrichiellaceae.g_Exophiala	4.14	3.71	0.00
p_Ascomycota.c_Sordariomycetes.o_Chaetosphaeriales	4.08	3.72	0.00
p_Ascomycota.c__Eurotiomycetes.o__Eurotiales.f__Aspergillaceae.g_Penicillium	4.61	4.10	0.00
p_Ascomycota.c_Eurotiomycetes.o_Chaetothyriales.f_Herpotrichiellaceae	4.24	3.81	0.00
p_Ascomycota.c_Sordariomycetes.o_Sordariales.f_Chaetomiaceae.g_Trichocladium	4.48	4.08	0.00
p_Mortierellomycota.c_Mortierellomycetes.o_Mortierellales.f_Mortierellaceae	4.84	4.37	0.00
p__Ascomycota.c__Leotiomycetes.o__Helotiales	4.35	3.96	0.00
p__Mortierellomycota	4.84	4.37	0.00
p_Ascomycota.c_Sordariomycetes.o_Chaetosphaeriales.f_Chaetosphaeriaceae.g__Chloridium	4.07	3.71	0.00

[1] (Fungi, LDA > 3.5)
