# Supplementary material for: Effects of Difenoconazole and Imidacloprid Seed Coatings on Soil Microbial Community Diversity and Ecological Function
Source: Microorganisms. 2025 Apr 1;13(4):806. doi: 10.3390/microorganisms13040806 (PMC12029232; doi:10.3390/microorganisms13040806)
Supplement: Supplementary file 1 [file microorganisms-13-00806-s001.zip › Table S4.pdf]

Table S4. Abundance Information of Wheat Rhizosphere Bacterial KEGG Pathways under Different Seed Coating Agent Treatments

KEGG Pathway Level2	Abundance				
	CK	D1	D1.5	I1	I1.5
Amino acid metabolism	7256673±153637 a	7118615±85883 b	7360564±151564 a	7163082±134142 b	7159583±70020 b
Biosynthesis of other secondary metabolites	1375167±20344 bc	1357852±17866 c	1407757±10393 a	1369062±3562 bc	1379274±13240 b
Carbohydrate metabolism	8135689±101731 b	8011875±110656 c	8317365±79600 a	8082431±65446 bc	8101170±40649 bc
Cell growth and death	783259±32838 a	772631±32763 a	785538±24439 a	773148±24024 a	780951±16596 a
Cell motility	805601±64604 c	804390±76077 c	964865±51844 a	876328±51590 b	909981±23284 b
Cellular community - prokaryotes	2049325±61656 bc	2005040±75608 c	2156635±54096 a	2066601±64830 b	2093590±39959 b
Energy metabolism	3943684±70705 a	3894438±28338 b	3982746±46709 a	3912713±46126 b	3931347±32825 b
Folding, sorting and degradation	1210742±11355 a	1198926±9079 b	1212036±9846 a	1198948±9738 b	1199623±4045 b
Glycan biosynthesis and metabolism	1055889±12586 a	1039489±8516 b	1060784±9814 a	1037137±8129 b	1047892±15737 a
Lipid metabolism	2055865±60379 b	2015625±65421 b	2111053±41745 a	2040242±44524 b	2047598±16301 b
Membrane transport	2519541±101900 bc	2435665±134917 c	2659484±86807 a	2507161±95935 bc	2531247±53000 b
Metabolism of cofactors and vitamins	3734868±77577 b	3687917±80543 b	3823488±59462 a	3724570±62567 b	3742783±35114 b
Metabolism of other amino acids	1422787±42263 b	1401232±41237 b	1458655±31860 a	1408848±27129 b	1419205±20997 b
Metabolism of terpenoids and polyketides	1005282±21853 a	986736±25004 a	1007847±23699 a	986904±21096 a	984575±8636 a
Nucleotide metabolism	2080751±28164 b	2051574±26802 c	2123792±28681 a	2063628±17302 bc	2065391±14404 bc
Replication and repair	2151339±26553 b	2135638±32796 b	2205677±32210 a	2149832±17584 b	2145251±15657 b
Signal transduction	2191250±100542 cd	2158910±111971 d	2364970±70493 a	2233998±86794 bc	2267418±35886 b
Signaling molecules and interaction	415±82 a	370±30 ab	316±121 b	340±86 b	310±61 b
Transcription	117598±1316 bc	117113±1599 c	121995±1450 a	118851±1868 b	118796±542 b
Translation	2472324±10463 a	2453725±9862 b	2450675±15646 b	2443311±4527 b	2438333±1033 b
Transport and catabolism	266919±11851 b	256475±12518 c	278956±7695 a	261494±7441 bc	263378±3526 bc
Xenobiotics biodegradation and metabolism	1750479±89876 b	1716149±99816 b	1835164±81120 a	1746310±69415 b	1770457±45058 ab

[1] The lowercase letters indicate significant differences between columns..
