# Supplementary figures and images for: Effects of Difenoconazole and Imidacloprid Seed Coatings on Soil Microbial Community Diversity and Ecological Function
Source: Microorganisms. 2025 Apr 1;13(4):806. doi: 10.3390/microorganisms13040806 (PMC12029232; doi:10.3390/microorganisms13040806)

**Figure S1. The relative abundance at the phylum level of fungi on day 120**

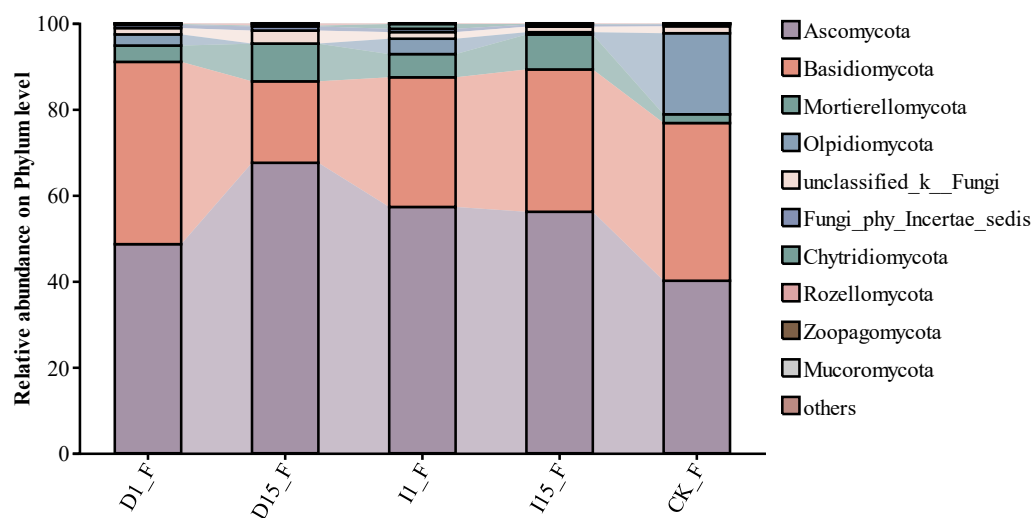

Supplement: Supplementary file 1 [file microorganisms-13-00806-s001.zip › Figure S1.pdf]
